# Milk-Derived Tripeptides IPP (Ile-Pro-Pro) and VPP (Val-Pro-Pro) Promote Adipocyte Differentiation and Inhibit Inflammation in 3T3-F442A Cells

**DOI:** 10.1371/journal.pone.0117492

**Published:** 2015-02-25

**Authors:** Subhadeep Chakrabarti, Jianping Wu

**Affiliations:** Department of Agricultural, Food & Nutritional Science (AFNS) and the Cardiovascular Research Centre (CVRC), University of Alberta, Edmonton, AB, Canada; Tohoku University, JAPAN

## Abstract

Milk derived tripeptides IPP (Ile-Pro-Pro) and VPP (Val-Pro-Pro) have shown promise as anti-hypertensive agents due to their inhibitory effects on angiotensin converting enzyme (ACE). Due to the key inter-related roles of hypertension, chronic inflammation and insulin resistance in the pathogenesis of metabolic syndrome, there is growing interest in investigating established anti-hypertensive agents for their effects on insulin sensitivity and inflammation. In this study, we examined the effects of IPP and VPP on 3T3-F442A murine pre-adipocytes, a widely used model for studying metabolic diseases. We found that both IPP and VPP induced beneficial adipogenic differentiation as manifested by intracellular lipid accumulation, upregulation of peroxisome proliferator-activated receptor gamma (PPARγ) and secretion of the protective lipid hormone adiponectin by these cells. The observed effects were similar to those induced by insulin, suggesting potential benefits in the presence of insulin resistance. IPP and VPP also inhibited cytokine induced pro-inflammatory changes such as reduction in adipokine levels and activation of the nuclear factor kappa B (NF-κB) pathway. Taken together, our findings suggest that IPP and VPP exert insulin-mimetic adipogenic effects and prevent inflammatory changes in adipocytes, which may offer protection against metabolic disease.

## Introduction

Metabolic syndrome, the presence of at least 3 of the following 5 clinical features such as hypertension (persistent elevation of blood pressure above 140/90 mm Hg), abdominal obesity, increased fasting serum glucose, raised serum triglycerides and low serum high density lipoprotein levels, is a growing problem worldwide [1,2]. People with metabolic syndrome are at significantly higher risks of developing atherosclerosis and type II diabetes which are major contributors to the global burden of morbidity and mortality [3]. Hypertension and diabetes/insulin resistance are the 2 major features of metabolic syndrome, which has led to the interest in developing novel therapies that may target the underlying pathologies common to both conditions [2,4]. Insulin resistance at the cellular level and persistence of chronic inflammation have been suggested as a key molecular mechanism contributing to the development of metabolic syndrome [5,6]. Not surprisingly, there is much interest in testing established anti-hypertensive therapies for their potential insulin-sensitizing functions which may improve the metabolic parameters as additional benefits, providing a dual protection against the key features of metabolic syndrome [7–9]. Indeed, recent evidence suggests that anti-hypertensive drugs targeting the renin-angiotensin system (such as angiotensin converting enzyme (ACE) inhibitors and angiotensin II receptor blockers) may exert beneficial effects on the metabolic system, involving modulation of insulin actions and suppression of chronic inflammation [10–12].

While insulin has wide ranging functions in different tissues and organs, its effects on the body’s fat cells or adipocytes play key roles in metabolic function in health and disease [13]. Under normal conditions, insulin promotes adipocyte differentiation involving incorporation of neutral lipids (such as triglycerides) into these cells, upregulation of the anti-inflammatory metabolic modulator peroxisome proliferator-activated receptor gamma (PPARγ) and secretion of the beneficial adipokine (hormone from fat tissue) adiponectin [14–17]. Under pathological conditions such as metabolic syndrome, these normal effects of insulin are perturbed, leading to abnormal adipogenesis as characterized by lack of differentiation in adipocyte precursors (pre-adipocytes), increased inflammation in such cells and higher levels of circulating lipids in the bloodstream [18–21]. Hence, compounds that can induce adipogenic differentiation in pre-adipocytes may improve insulin sensitivity and/or act as insulin-mimetics which would potentially improve the management of conditions such as metabolic syndrome [22–24].

Despite advances in the treatment of conditions like hypertension and type II diabetes, pharmacological management of these conditions often requires lifelong therapy and can have significant side-effects [25,26]. As such, there is growing interest in developing natural product based therapies against chronic diseases such as metabolic syndrome which require lifelong adherence to therapeutic regimens. Health care products derived from food sources are widely accepted because of their intake in diet and perceived lack of serious side-effects. Indeed, a number of bioactive peptides and enzymatic hydrolysates isolated from food-derived proteins have shown promise in managing features associated with metabolic syndrome to date [27–29].

Milk is a source of food proteins that can generate beneficial bioactive products on enzymatic breakdown [30,31]. Hydrolysis of the milk protein casein yields 2 important bioactive tripeptides with ACE-inhibitor activity [32]. These peptides, IPP (Ile-Pro-Pro) and VPP (Val-Pro-Pro) have been shown to improve management of hypertension (in both laboratory animals and clinical trials of humans) as well as exhibit anti-inflammatory effects which make these attractive targets for managing metabolic syndrome [33–36]. However, whether or not these peptides can mimic insulin actions or improve adipocyte functions remain to be determined.

Given this background, we investigated the roles of milk derived tripeptides IPP and VPP on adipogenic differentiation and modulation of inflammatory changes in a widely used pre-adipocyte cell culture system. Our findings indicate presence of insulin-mimetic and anti-inflammatory actions in both peptides which may have potential applications in the management of metabolic syndrome.

## Materials and Methods

### Bioactive peptides

The tripeptides IPP and VPP were both purchased from Genscript (Piscataway, NJ, USA). The peptides were dissolved in sterile phosphate buffered saline (1XPBS), aliquoted and stored at -20°C until the time of experiments. Peptide sequences were validated by MS/MS while the purity of both peptides was determined by HPLC (99.9% for IPP and 98.6% for VPP) according to the manufacturer.

### Reagents

Dulbecco’s phosphate buffered saline (PBS), LipidTox dye and dithiothreitol (DTT) were all from Sigma Aldrich (St Louis, MO, USA). Dulbecco’s modified Eagle medium (DMEM) and fetal bovine serum (FBS) were purchased from Gibco/ Invitrogen (Carlsbad, CA, USA). Type 1 Collagenase used for cell splitting was from Worthington Biochemical Corporation (Lakewood, NJ, USA). The murine tumor necrosis factor alpha (TNFα) was obtained from Peprotech (Rocky Hill, NJ, USA). Triton-X-100 was from VWR International (West Chester, PA, USA).

### Cell culture & adipogenic differentiation

The commercially available murine pre-adipocyte cell line 3T3-F442A (Sigma Aldrich; Cat# 00070654) was used for all experiments. The frozen cells were obtained in passage 8, thawed and expanded in culture using DMEM supplemented with 10% FBS (heat-inactivated) and antibiotics. The cells were grown in T-25 flasks to confluence prior to sub-culture in gelatin-coated 48 well plates for the actual experimental procedures. All studies were performed using cells in passages 10–22.

To determine the ability of tripeptides to induce adipogenic differentiation, the cells (grown in 48 well plates) were incubated in standard culture medium (DMEM + 10% FBS + antibiotics) in the presence of insulin or the tripeptides of interest for 72 hr without changing the medium. Insulin was used as a positive control. Adipogenic changes were determined by the appearance of intracellular lipid droplets (as shown by LipidTox staining), upregulation of PPARγ (shown by western blot) and release of the beneficial adipokine, adiponectin (measured by ELISA). Insulin (10 μg/mL) was used as a positive control for inducing differentiation.

To study the effects of TNFα stimulation on adiponectin release, insulin (10 μg/mL) treatment was carried out for 48 hr followed by incubation in a resting/quiescing medium (DMEM + 1% FBS + antibiotics) for a further 24 hr in the presence/absence of TNFα (10 ng/mL). For some experiments, the tripeptide of interest was added to the resting medium 1 hr prior to addition of TNFα.

### Intracellular lipid staining

Increase in intracellular accumulation of lipid droplets is a hallmark of adipogenic differentiation [37]. To detect this, 3T3-F442A cells were first grown to 60–70% confluence prior to changing the culture medium (DMEM + 10% FBS + antibiotics) and addition of either IPP or VPP (both 50 μM). After 72 hr incubation with IPP or VPP, the cells were fixed and stained for neutral lipid by the cell-permeable dye LipidTox (dissolved in 1XPBS, 1:250 dilution) for a 30 min period. The cells were then counter-stained with the Hoechst33342 (Molecular Probes, Eugene, OR, USA) nuclear dye (1:10,000 dilution in 1XPBS), washed 3 times in buffer and visualized under an Olympus IX81 fluorescent microscope (Carson Scientific Imaging Group; Markham, Ontario, Canada). Images were obtained and analyzed using the Metamorph imaging software (Molecular Devices, Sunnyvale, CA) and presented at (200X) magnification. A control image from a group of cells without LipidTox treatment was used to detect any nonspecific fluorescence. The images were then quantified by subtracting the background fluorescence of the control image, so only fluorescence from the lipid-specific staining was visible. The fluorescence intensity was then measured for quantitative analysis.

### Biochemical lipid assay

The intracellular lipid content was also determined by a commercially available biochemical assay (Adipogenesis colorimetric assay kit; BioVision, Milpitas, CA, USA) as per the manufacturer’s instructions. Briefly, the cells were lysed by boiling hot assay buffer to release the lipids, which were converted by exogenous lipase into glycerol and fatty acid prior to being detected by colorimetric method in a reaction involving a chemical probe and the requisite enzyme mix.

### Western blotting

At the end of the experimental intervention/s, culture medium was removed and the cells lysed in boiling hot Laemmle’s buffer containing 50 μM dithiothreitol (DTT, a reducing agent) and 0.2% Triton-X-100 to prepare samples for western blotting (similar to our previous study [38]). These cell lysates were then run in SDS-PAGE, transferred to nitrocellulose membranes and immunoblotted with antibodies against c-Jun (mouse monoclonal antibody from EMD Millipore cat# 05-1076; Billerica, MA, USA), CCAAT/ enhancer binding protein alpha (C/EBPα; rabbit polyclonal antibody from Cell Signaling Technology, cat# 2295; Boston, MA, USA), peroxisome proliferator-associated receptor gamma (PPARγ; rabbit polyclonal antibody from Cell Signaling Technology, cat# 2430), inhibitor κBα (IκBα; rabbit polyclonal antibody from Cell Signaling Technology, cat# 9242), phospho-p65 (rabbit polyclonal antibody from Santa Cruz Biotechnology, cat# sc-3033; Santa Cruz, CA, USA), p65 (mouse monoclonal antibody from Santa Cruz Biotechnology, cat# sc-8008) and the loading control α-tubulin (rabbit polyclonal antibody from Abcam, cat# ab15246; Cambridge, MA, USA). Anti-tubulin was used at 0.4 μg/mL, while all others were used at 1 μg/mL. Goat anti-rabbit and Donkey anti-mouse fluorochrome-conjugated secondary antibodies were from Licor Biosciences (Lincoln, NB, USA). The protein bands were detected by a Licor Odyssey BioImager and quantified by densitometry using corresponding software (Licor Biosciences). Each band of IκBα, PPARγ, C/EBPα or c-Jun was normalized to its corresponding band of loading control while phospho-p65 bands were compared to their corresponding total p65 bands. Cell lysates from untreated cells were loaded onto every gel for comparison.

### Estimation of adiponectin

The culture media were collected from 3T3-F442A cells grown in 48 well plates at the end of experimental procedures. The media were centrifuged (10,000Xg for 10 min at 4°C) to yield cell-free supernatants which were stored at -80°C until the time of the assay. Cell-free supernatants were thawed and used in the commercially available Mouse Adiponectin DuoSet ELISA kit (R&D Systems; Minneapolis, MN, USA) as per the manufacturer’s instructions. Data were expressed as percentage of supernatants from the untreated cells.

### Statistical analysis

All data are presented as mean ± SEM (standard error of mean) from between 4 and 6 independent experiments. One way analysis of variance (ANOVA) was used for determination of statistical significance, with the appropriate post-hoc test (Dunnett’s test for comparison to control group; Tukey’s test for multiple comparisons). Repeated measures test was used wherever applicable. The PRISM 5 statistical software (Graph Pad Software, San Diego, CA) was used for the analyses. P< 0.05 was considered to be significant.

## Results

### IPP and VPP enhance intracellular lipid accumulation in 3T3-F442A cells

**Fig 1:**
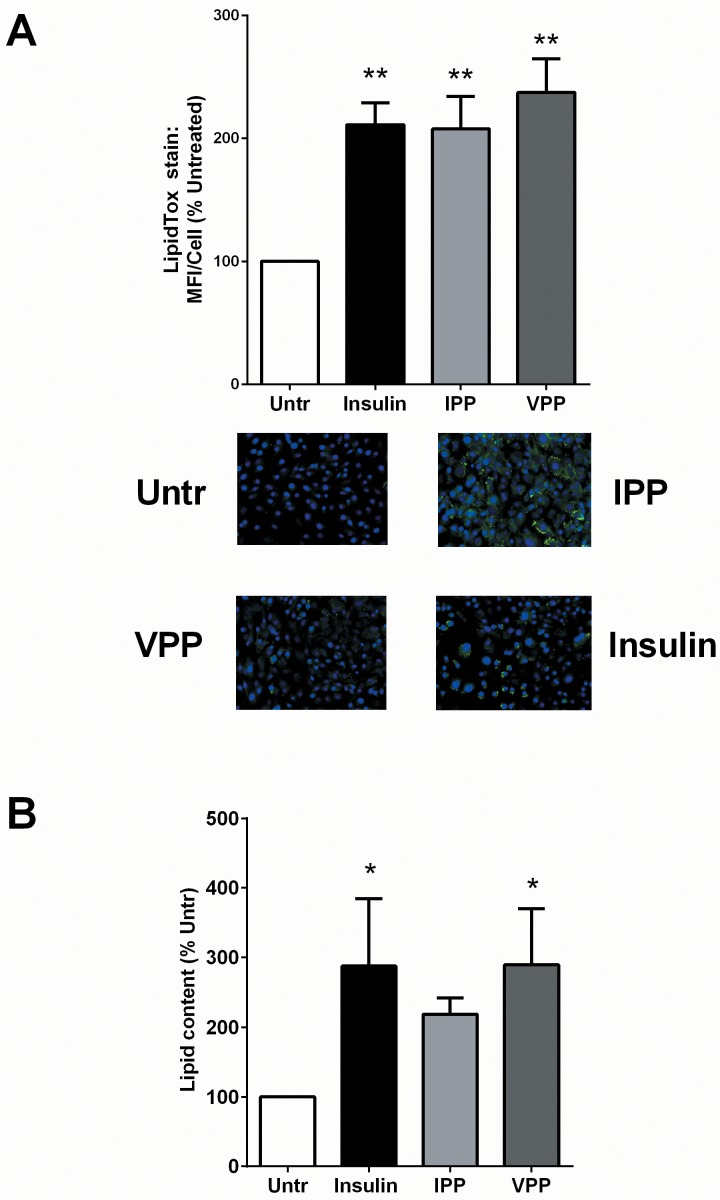
IPP and VPP induce lipid accumulation in 3T3-F442A cells. 3T3-F442A cells were incubated in presence of insulin (10 μg/mL), IPP (50 μM) or VPP (50 μM) for 72 hr. (A) For one study, the cells were fixed, stained with the neutral lipid-specific dye LipidTox and visualized under fluorescence microscopy. A set of representative images are shown. (B) For another set of experiments, the cells were lysed and their lipid contents were estimated by a biochemical assay. Data were presented as mean±SEM of 3–4 independent experiments. * and ** indicate p<0.05 and p<0.01 respectively compared to the untreated control (Untr).

Both IPP and VPP treatment (50 μM, 72 hr) significantly increased intracellular lipid accumulation, suggesting enhanced adipogenic differentiation in the pre-adipocytes (Fig. 1A). Insulin (10 μg/mL) was used as a positive control for these studies and it showed a similar effect. These findings were further validated by a quantitative biochemical assay for cellular lipid content which demonstrated significant increases in intracellular lipid levels upon incubation with both insulin and VPP. Treatment with IPP also appeared to increase lipid content although it did not reach statistical significance (Fig. 1B).

Neither IPP nor VPP at the given concentration was cytotoxic to the 3T3-F442A cells after 72 hr incubation, as determined by Trypan blue staining (Trypan blue positive cells per field (under light microscopy; X100): 5.668±0.385 for untreated, 6.557±0.676 for IPP-treated and 4.445±0.112 for VPP-treated groups).

### IPP and VPP upregulate the markers of adipogenic differentiation in 3T3-F442A cells

**Fig 2:**
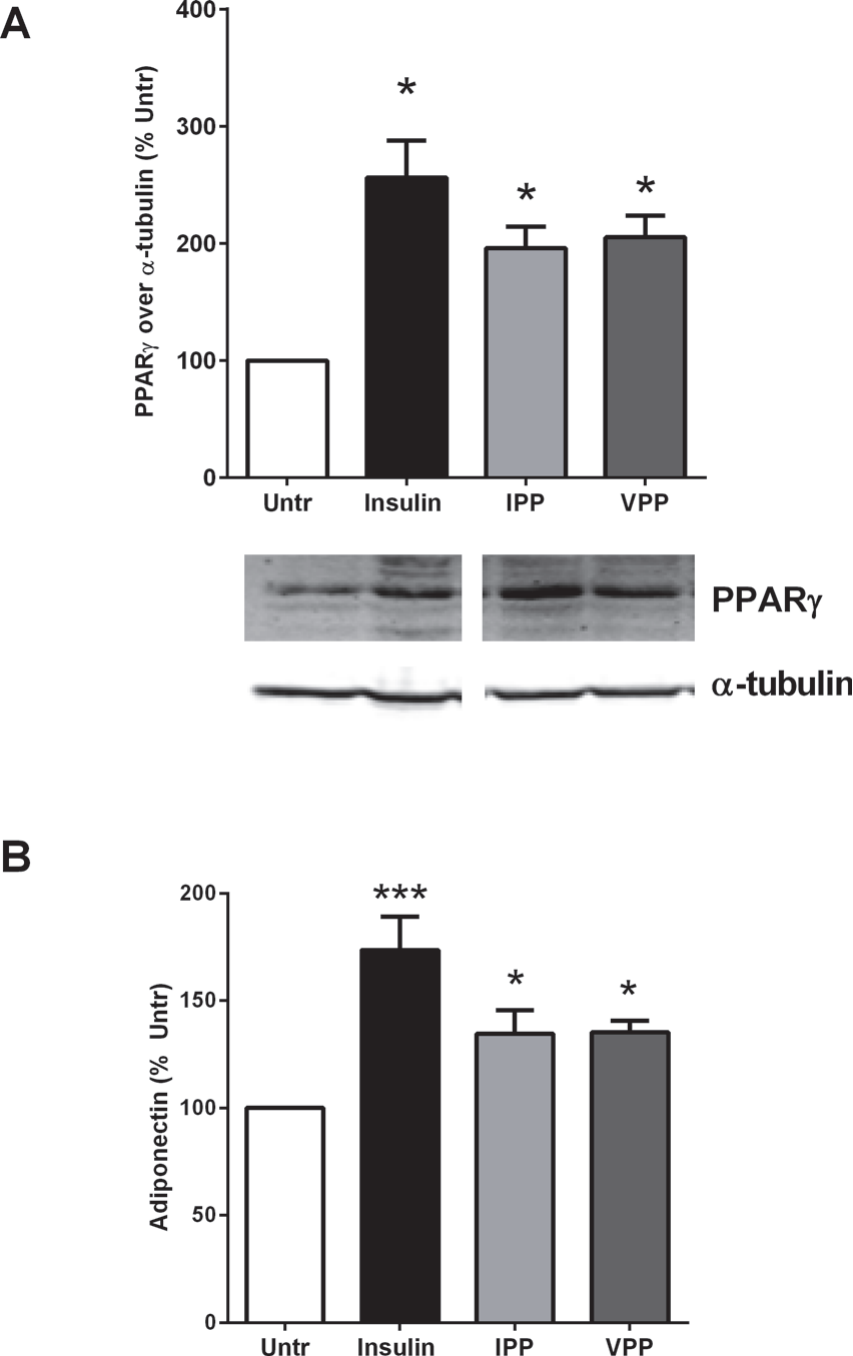
IPP and VPP promote expression of the adipocyte differentiation markers PPARγ and adiponectin in 3T3-F442A cells. 3T3-F442A cells were incubated in presence of insulin (10 μg/mL), IPP (50 μM) or VPP (50 μM) for 72 hr. (A) The cells were lysed and western blotting of the lysates was performed with antibodies against PPARγ and α-tubulin (loading control). A set of representative images (including cropped images obtained from the same membrane) was shown. (B) The cell-free culture supernatants were collected and analyzed by ELISA to determine levels of adiponectin. Data were presented as mean±SEM of 5 independent experiments. * and *** indicate p<0.05 and p<0.001 compared to the untreated control (Untr) respectively.

Adipocyte differentiation is regulated by signaling through PPARγ and associated with increased synthesis and release of the beneficial adipokine, adiponectin. Hence, upregulation of both PPARγ and adiponectin are widely used as markers for adipogenic changes [39–41]. We found that 72 hr incubation with either IPP or VPP significantly increased PPARγ expression in cell lysates of 3T3-F442A cells (Fig. 2A). These changes were associated with concomitant increases in adiponectin secretion into the culture media (Fig. 2B). Insulin (10 μg/mL), used as a positive control, also mirrored the changes induced by IPP and VPP (Fig. 2), indicating successful induction of adipogenic differentiation in this system. As the effects of IPP and VPP on upregulation of differentiation markers were comparable to those exerted by insulin, these tripeptides appear to be promising as insulin mimetic agents on adipocyte behaviour.

### IPP and VPP increase expression of transcriptional regulators for adipogenic differentiation

**Fig 3:**
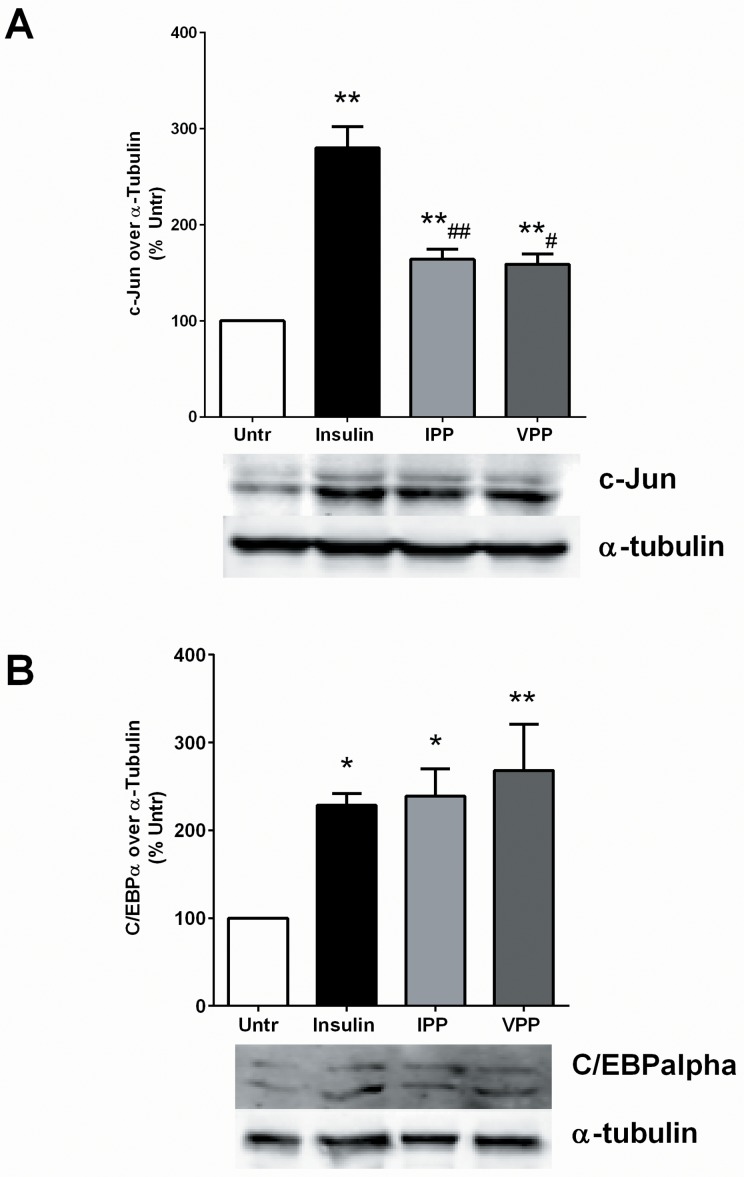
IPP and VPP increase the protein levels of adipocyte differentiation regulators c-Jun and C/EBPα. 3T3-F442A cells were incubated in presence of insulin (10 μg/mL), IPP (50 μM) or VPP (50 μM) for 72 hr. The cells were lysed and western blotting of the lysates was performed with antibodies against c-Jun (A), C/EBPα (B) and α-tubulin (loading control). A set of representative images was shown. Data were presented as mean±SEM of 4–6 independent experiments. * and ** indicate p<0.05 and p<0.01 respectively, compared to the untreated control (Untr). # and ## indicate p<0.05 and p<0.01 respectively, compared to the insulin treated cells.

The adipogenic differentiation is a complex but highly regulated phenomenon depending on transcriptional upregulation of a number of different molecules. Transcriptional regulators such as c-Jun and C/EBPα play critical roles in mediating a series of co-ordinated events that result in successful differentiation as mature adipocytes [14,42–44]. Indeed, compounds capable of adipogenesis are known to upregulate these molecules in pre-adipocytes and consequently promote their differentiation [44–46]. Given the ability of IPP and VPP to induce adipogenic changes, we tested their ability to upregulate c-Jun and C/EBPα levels in our system. We found that both IPP and VPP significantly increased protein levels of both c-Jun and C/EBPα, as did the addition of exogenous insulin (Fig. 3A and B). However, the degree of c-Jun upregulation by insulin was significantly greater than that induced by the 2 tripeptides while the effects on C/EBPα upregulation were comparable between insulin and the peptides. Our results indicate that IPP and VPP induce adipogenic effects at least partially involving c-Jun and C/EBPα dependent pathways.

### Pre-treatment with IPP or VPP attenuates TNFα mediated activation of pro-inflammatory transcriptional factor NF-κB

**Fig 4:**
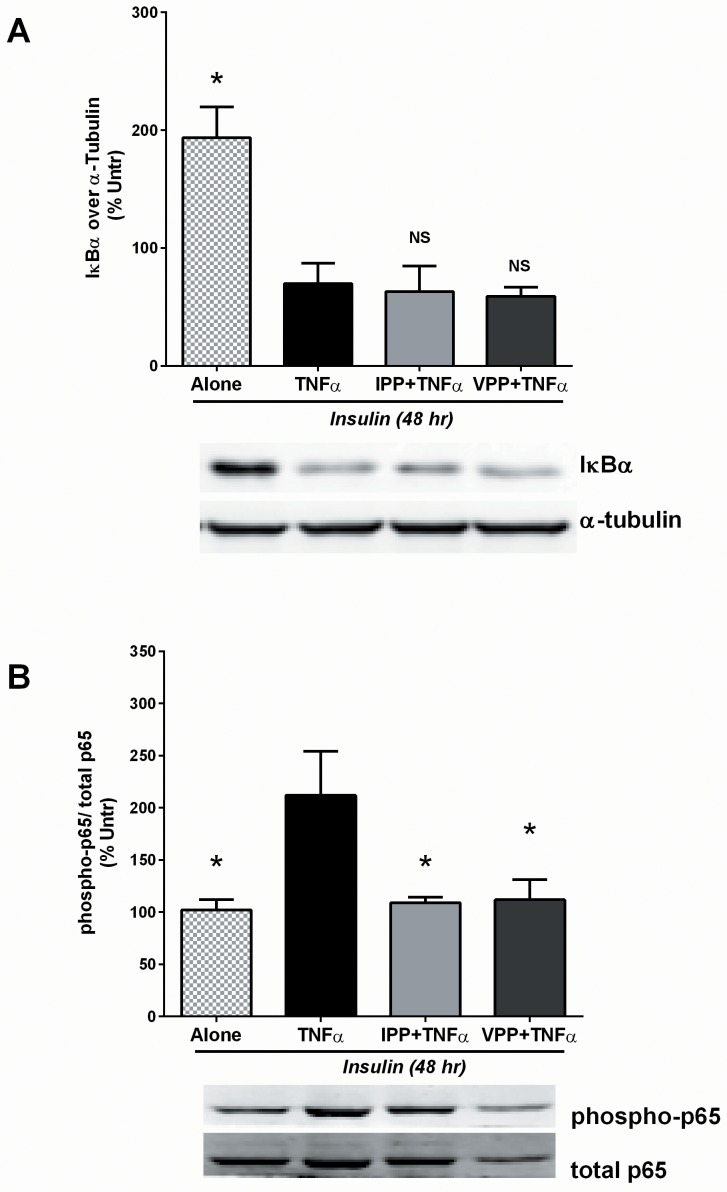
IPP and VPP inhibit TNFα mediated activation of the pro-inflammatory NF-κB pathway downstream of IκB degradation. 3T3-F442A cells were incubated for 48 hr in presence of insulin (10 μg/mL) to induce differentiation. Cells were then washed and further incubated for 30 min with pro-inflammatory cytokine TNFα (10 ng/mL) with/without addition of IPP (50 μM) or VPP (50 μM). Afterwards, the cells were lysed and western blotting of the lysates was performed to determine (A) IκBα degradation (using antibodies against IκBα and the loading control, α-tubulin) and (B) p65 phosphorylation (using antibodies against phosphorylated and total p65). A set of representative images was shown. Data were presented as mean±SEM of 4 independent experiments. All data were normalized to the values from untreated (i.e. undifferentiated) cells. * indicates p<0.05 compared to the TNFα treated cells. NS means: not significant (compared to TNFα treated group).

Next we evaluated the effect of these tripeptides on inflammatory changes in differentiated adipocytes using the pro-inflammatory cytokine, TNFα. TNFα has widespread inflammatory effects in the body and has been implicated in harmful changes in adipocytes such as activation of the pro-inflammatory nuclear factor-kappaB (NF-κB) pathway and susceptibility to metabolic syndrome [47,48]. 3T3-F442A cells were first treated with insulin (48 hr) and then treated for a further 24 hr in the presence of IPP or VPP as described in Materials and Methods section. The cells were then stimulated with TNFα (10 ng/mL) for 30 min. TNFα induced degradation of IκBα (needed for release of p65 and p50 components) and phosphorylation of p65 (which is needed for its nuclear translocation and target binding) were examined as measures of NF-κB activation. We found that pre-treatment with neither IPP or VPP could prevent TNFα-induced degradation of IκBα (Fig. 4A), while both peptides were able to attenuate the increased p65 phosphorylation (Fig. 4B). These data suggest that both IPP and VPP are able to modulate NF-κB activity, likely at a site downstream of IκBα and involving p65 phosphorylation.

### IPP or VPP pre-treatment prevents TNFα induced loss of adiponectin release from 3T3-F442A cells

**Fig 5:**
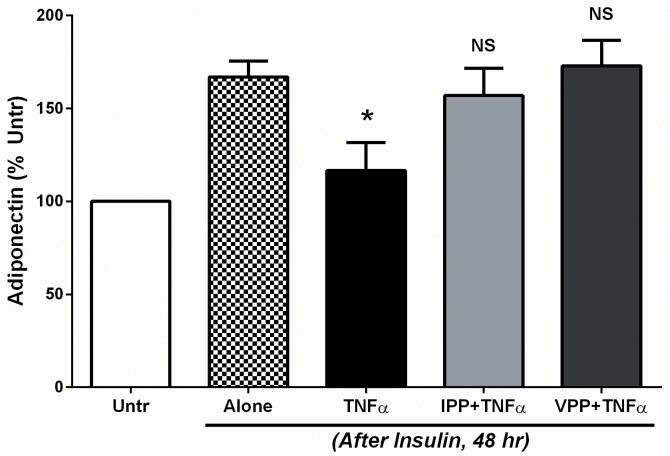
IPP and VPP prevent TNFα mediated loss of adiponectin release from insulin-differentiated 3T3-F442A cells. 3T3-F442A cells were incubated for 48 hr in presence of insulin (10 μg/mL) to induce differentiation. Cells were then washed and further incubated for 24 hr with the pro-inflammatory cytokine TNFα (10 ng/mL) with/without addition of IPP (50 μM) or VPP (50 μM). At the end of this incubation period, the cell-free supernatants were collected and analyzed for their adiponectin content by ELISA. Data were presented as mean±SEM of 4 independent experiments. * indicates p<0.05 compared to the insulin alone (Alone). NS means: not significant (compared to Alone).

Inflammatory mediators such as TNFα are known to inhibit adipokine secretion and consequently contribute towards metabolic syndrome [49]. Insulin treated (48 hr) 3T3-F442A cells were then used to examine TNFα induced alterations in adipokine release. Cells were treated with TNFα (10 ng/mL) in presence/absence of IPP or VPP. A 24 hr incubation with TNFα caused significant reduction in levels of secreted adipokine, bringing these to the levels observed in undifferentiated cells (Fig. 5). Presence of either tripeptide completely prevented the TNFα mediated suppression of adipokine release (Fig. 5), demonstrating a beneficial anti-inflammatory effect in cytokine activated adipocytes.

## Discussion

The major findings from this study are: (i) IPP and VPP induce adipogenic differentiation in murine pre-adipocytes similar to insulin; (ii) these actions involve upregulation of transcriptional regulators such as c-Jun and C/EBPα; (iii) both tripeptides also suppress cytokine mediated inflammatory effects in differentiated adipocytes, involving modulation of adipokine release and NF-κB activation.

Insulin is a key anabolic hormone in the metabolic system. Insulin mediates the uptake and utilization of glucose in various tissues in addition to its anti-inflammatory and adipogenic roles. Loss of insulin sensitivity in its target organs such as adipocytes, liver or skeletal muscle leads to improper storage and utilization of glucose and lipids which contribute towards the pathogenesis of both type II diabetes and metabolic syndrome [2,50]. The causes of insulin resistance are incompletely understood, but chronic inflammation, aging and stress all appear to play some roles [49]. The lack of insulin responses in adipocytes is a key pathogenic mechanism underlying metabolic syndrome [18]. In the absence of insulin actions, circulating levels of lipids and lipoproteins are raised while adipose tissues (the normal reservoirs for the body’s lipids) are poorly developed. In addition, impaired differentiation of the adipocytes predisposes these cells to inflammatory insults and further contributes to the pathological state [6]. For example, normally differentiated adipocytes release adiponectin, a beneficial adipokine with anti-inflammatory and anti-atherogenic profile [14]. Inflamed and abnormally differentiated adipocytes are, on the other hand, a source for pro-inflammatory cytokines (e.g. TNFα, interlukin-8) and adipokine (e.g. leptin) which predispose to atherosclerosis and further aggravation of insulin resistance and diabetes [51]. Hence, restoration of insulin actions, either by improving insulin sensitivity or by the use of compounds mimicking insulin, has been a goal for controlling metabolic syndrome and its complications. Several different commercially available cell lines have been used for studying adipocyte functions including the murine adipocytes, 3T3-L1 and 3T3-F442A [52,53]. While 3T3-L1 cells have been used more widely, they require not only insulin but also glucocorticoids for inducing adipogenic differentiation which makes these less suitable for testing insulin-like properties of exogenous compounds. In contrast, insulin alone is sufficient to induce 3T3-F442A cells to develop into mature adipocytes, indicating these cells are more differentiated and consequently better suited for examining the adipogenic potential of novel substances, as in the present study [53].

Given the role of impairments in the renin-angiotensin system (RAS) in the pathogenesis of hypertension and metabolic syndrome, recent research has also focussed on the potential beneficial effects of drugs targeting RAS pathway on other aspects of metabolic diseases [11]. Indeed, ACE inhibitors such as captopril have been demonstrated to improve adipocyte differentiation and exert anti-inflammatory effects in various tissues, suggesting their additional benefits in a complex condition like metabolic syndrome [54]. However, whether these effects are due to their actions on RAS or incidental ‘off-target’ benefits is still not clear. Both IPP and VPP were originally identified based on their ACE inhibitor actions which were further validated by their anti-hypertensive effects in laboratory animals and human studies [34]. Indeed, commercial fermented milk preparations enriched in IPP and VPP have been promoted for improving cardiovascular functions [55–57]. There is also growing evidence of anti-inflammatory actions of IPP and VPP as shown by both cell based and animal research [55,58]. However, the roles of IPP and/or VPP on metabolism and insulin signaling have not been explored before. Results from our study have now demonstrated that IPP and VPP can indeed promote insulin-like differentiating effects on pre-adipocytes. Due to the concomitant presence of hypertension, inflammation and insulin resistance in many cases of metabolic syndrome, these tripeptides may provide a novel naturally based option for its management.

Inflammation is another key factor in the development, persistence and complications of metabolic syndrome [20]. While abnormal RAS activity and insulin resistance (such as impaired insulin effects on pre-adipocytes) can contribute to inflammatory changes, chronic inflammation by itself is also a causative factor in developing insulin resistance [59]. Indeed, inflammation in the adipocytes, leading to release of harmful cytokines and adipokines, is a major factor underlying the altered metabolic state that characterizes metabolic syndrome and its complications [60]. On the other hand, insulin mediated actions on adipocytes such as upregulation of adiponectin and PPARγ are known to exert anti-inflammatory effects [61]. For example, PPARγ activation results in anti-inflammatory and anabolic actions; hence, PPARγ agonists like rosiglitazone have been used in the management of type II diabetes, a disease caused by widespread resistance to insulin actions [62,63]. Our studies demonstrated upregulation of both PPARγ and adiponectin by IPP and VPP, suggesting their contribution to enhancing these vital anti-inflammatory pathways. In addition, IPP and VPP further inhibited inflammatory signaling by TNFα, a major pro-inflammatory cytokine involved in the pathology of atherosclerosis and other inflammatory diseases. TNFα is known to contribute towards the pathology of metabolic syndrome and agents that could suppress TNFα effects have been touted as potential therapies against this condition [64]. For example, TNFα attenuates the release of adiponectin from adipocytes which further complicates the pathology of metabolic syndrome [65]. A number of agents with anti-inflammatory properties have evaluated for their ability to suppress these TNFα-mediated adverse effects on adipocytes, ranging from pharmacological antioxidants like beta-mercaptoethanol (BME) to natural compounds extracts from St John’s wort [66,67]. The common theme running through these studies is the ability of tested compound/s to successfully prevent the harmful TNFα effects and restore normal adipocyte functions. Our data suggests a prominent role for both IPP and VPP in preventing TNFα induced loss of adiponectin release, which may offer protection against perturbations of the metabolic state. The tripeptides also inhibited TNFα dependent activation of NF-κB, a major pro-inflammatory signaling mechanisms that is involved in upregulation of many proteins contributing towards inflammation and metabolic syndrome [68]. NF-κB activation involves the initial phosphorylation and degradation of IκB molecules, which releases the p65 and p50 components from the cytoplasm and facilitates their subsequent phosphorylation and nuclear translocation ultimately leading to the binding of p65/p50 homodimers to regulatory elements in the genes of inflammatory proteins (reviewed in [69]). Interestingly, we found that the tripeptides did not affect TNFα induced degradation of IκBα, while they significantly attenuated the phosphorylation of p65 which is known to interfere with nuclear translocation. These mechanisms are comparable to those previously identified by our group in case of an egg derived tripeptide (IRW) in cytokine treated endothelial cells where IRW prevented p65 nuclear translocation without affecting degradation of IκBα [70]. Whether the inhibition of NF-κB at a point downstream of IκB represent a common pathway for anti-inflammatory bioactive peptides remains to be determined.

The mechanisms of action of IPP and VPP are as yet incompletely understood. Both are rich in proline (Pro) and current research suggests that peptides with proline residues are likely to have anti-inflammatory and ACE inhibitory activities [71,72]. Proline-rich peptides are also less amenable to gastro-intestinal digestion and more likely to be absorbed upon oral consumption, a factor that can be beneficial for future therapeutic usage [73]. Despite this apparently beneficial profile, no specific receptor or unique signaling pathway has been identified to explain these beneficial biological effects. It is, however, likely that both peptides act through multiple pathways to mediate their actions on different aspects of adipocyte function. Whether or not these actions are independent of their well characterized ACE inhibition is also unknown. It is not implausible that insulin like actions of IPP and VPP on pre-adipocytes is due to their ACE inhibitory activities since pre-adipocytes are known to have a functioning RAS pathway which can be modulated for pharmacological benefit [74,75]. Alternatively, these differentiating effects could also be mediated independently of RAS, possibly by binding to insulin receptor and acting as complete or partial agonists. A third possibility is the existence of as yet undetermined novel signaling pathways stimulated by tripeptides like IPP and VPP which may or may not interact with other established signaling pathways such as those downstream of insulin, TNFα or angiotensin exposure. Delineating the molecular mechanisms of action underlying the beneficial effects of IPP and VPP is likely to be an important area of future research.

To summarize, we found that milk derived anti-hypertensive tripeptides IPP and VPP exert insulin-like activities on adipocytes and suppress cytokine mediated inflammatory responses in these cells. Given the critical roles of adipose tissue in its pathogenesis and the inter-related roles of hypertension, inflammation and insulin resistance; these tripeptides may have therapeutic potential in the management of metabolic syndrome and its complications.
